# Visible Light Photoactivity of g-C_3_N_4_/MoS_2_ Nanocomposites for Water Remediation of Hexavalent Chromium

**DOI:** 10.3390/molecules29030637

**Published:** 2024-01-30

**Authors:** Chunmei Tian, Huijuan Yu, Ruiqi Zhai, Jing Zhang, Cuiping Gao, Kezhen Qi, Yingjie Zhang, Qiang Ma, Mengxue Guo

**Affiliations:** 1 College of Agriculture and Biological Science, Dali University, Dali 671000, China; tiancm1985@hotmail.com (C.T.); hjyu_yhj@163.com (H.Y.); zhairuiqi2023@163.com (R.Z.); zj2452488891@163.com (J.Z.); gaocp_dlu@163.com (C.G.); 2 College of Pharmacy, Dali University, Dali 671000, China; qkzh2003@aliyun.com; 3 Key Laboratory of Ecological Microbial Remediation Technology of Yunnan Higher Education Institutes, Dali University, Dali 671000, China; 4 School of Architecture and Civil Engineering, Chengdu University, Chengdu 610106, China; 5 Resources and Environment Institute, Yunnan Land and Resources Vocational College, Kunming 652501, China; mxx921@163.com

**Keywords:** g-C_3_N_4_/MoS_2_ composites, Z-scheme heterojunction, hexavalent chromium Cr (VI), photocatalyst

## Abstract

Water pollution has becoming an increasingly serious issue, and it has attracted a significant amount of attention from scholars. Here, in order remove heavy metal hexavalent chromium (Cr (VI)) from wastewater, graphitic carbon nitride (g-C_3_N_4_) was modified with molybdenum disulfide (MoS_2_) at different mass ratios via an ultrasonic method to synthesize g-C_3_N_4_/MoS_2_ (CNM) nanocomposites as photocatalysts. The nanocomposites displayed efficient photocatalytic removal of toxic hexavalent chromium (Cr (VI)) from water under UV, solar, and visible light irradiation. The CNM composite with a 1:2 g-C_3_N_4_ to MoS_2_ ratio achieved optimal 91% Cr (VI) removal efficiency at an initial 20 mg/L Cr (VI) concentration and pH 3 after 120 min visible light irradiation. The results showed a high pH range and good recycling stability. The g-C_3_N_4_/MoS_2_ nanocomposites exhibited higher performance compared to pure g-C_3_N_4_ due to the narrowed band gap of the Z-scheme heterojunction structure and effective separation of photo-generated electron–hole pairs, as evidenced by structural and optical characterization. Overall, the ultrasonic synthesis of g-C_3_N_4_/MoS_2_ photocatalysts shows promise as an efficient technique for enhancing heavy metal wastewater remediation under solar and visible light.

## 1. Introduction

Water scarcity and water pollution have long been major global concerns; in recent decades, the rapid development of the economy and industrialization led to increasingly serious environmental pollution problems. Water quality has decreased since a large amount of industrial wastewater, which contained heavy metals, was discharged into the water system [1,2]. Chromium is one of the common sources of heavy metal pollution and mainly exists in Cr (III) and Cr (VI) in water [3]. Cr (III) is one of the essential elements in the human body, which can participate in the metabolism of human fat and is widely used in the adjuvant therapy of diabetes [4]. Cr (VI) poses a lasting threat to the environment and human health and can enter the human body through skin-to-skin contact or breathing. In addition, Cr (VI) has strong oxidation and can oxidize human hemoglobin into methemoglobin, which may cause cancer risk after long-term or short-term exposure [5,6]. Therefore, it is highly important that we find a way to handle Cr (VI) in industrial wastewater economically and efficiently and make it meet the discharge standard.

At present, reducing Cr (VI) to Cr (III) in wastewater is an important way of alleviating chromium pollution in water [7,8], and common methods for treating chrome-containing heavy metal wastewater include the adsorption method [9,10], chemical reduction method [11,12], and biological method [13,14]. In the process of Cr (VI) removal, these methods consume a large amount of power and other resources, have high costs, and may cause other forms of pollution. However, existing treatment methods for the removal of pollutants in the process of treatment have complexity and high costs, and they are prone to secondary pollution and other shortcomings.

Photocatalytic technology is widely used [15,16,17], which has the advantages of no secondary contamination and strong redox capacity and is widely used in pollutant degradation [18,19], hydrogen generation [20,21] and CO_2_ photoreduction [22]. It is one of the best ways to solve future pollution problems [23]. Bi-bridge S-scheme Bi_2_S_3_/BiOBr heterojunction (Bi_2_S_3_/Bi/BiOBr), produced by the one-pot solvothermal method, has shown high visible light photocatalytic reduction performance, and the removal efficiency of Cr (VI) was 97% [24]. Graphitic carbon nitride (g-C_3_N_4_)’s band gap is 2.7 eV; it is a common photocatalyst with high stability and environmental friendliness [25,26,27]. Currently, thermal polycondensation is commonly used in the preparatory work of g-C_3_N_4_, which makes the preparation of g-C_3_N_4_ simple [28,29,30]. However, due to the rapid recombination rate of photo-generated electrons and holes and the low light absorption range and surface range [31,32,33], the photocatalytic performance of g-C_3_N_4_ photocatalysts is low, which makes the use of g-C_3_N_4_ limited. Numerous researchers have found that the photocatalytic performance of g-C_3_N_4_ could be improved by conducting morphology regulation [34,35], ion doping [36,37,38], and heterojunction construction [39,40,41]. Li et al. [42] prepared ultra-thin tubular lateral heterostructures (LHSs) of graphitic carbon nitride and carbon dots (CN/C-Dots) by one-step thermal polymerization; they found that the CN/C-Dots LHSs exhibited excellent electrocatalysts for a hydrogen evolution reaction, due to which the charge carriers’ transport was enhanced and the specific surface area was increased, meaning more active sites of CN. Renji Rajendran et al. [43] developed a g-C_3_N_4_/TiO_2_/α-Fe_2_O_3_ ternary magnetic nanocomposite with a Z-scheme by facile calcination and a hydrothermal process. The g-C_3_N_4_/TiO_2_/α-Fe_2_O_3_ ternary magnetic nanocomposite exhibited excellent photocatalytic performance for the degradation of Rhodamine B (RhB); under visible light exposure, the degradation rate was 95.7%, which was due to the formation of the Z-scheme enhancing the separation and migration of photoexcited electron and hole pairs and the light absorption range. Photocatalysts have been widely used for various purposes. However, photocatalysts still have the shortcomings of low utilization of visible light and high requirements for reaction conditions. MoS_2_ has attracted attention as a transition metal dichalcogenide with good chemical stability and adjustable bandwidth [44].

In this work, g-C_3_N_4_/MoS_2_ samples with different mass ratios were prepared by the ultrasonic method, and the photocatalytic removal efficiencies of Cr (VI) under different light irradiation sources (ultraviolet light, solar light and visible light) were investigated. g-C_3_N_4_/MoS_2_ composites showed strong photocatalytic activity. When the pH value was 3, the initial concentration of Cr (VI) was 20 mg/L, and the photocatalyst demonstrated strong photocatalytic activity. Compared with pure g-C_3_N_4_, the doping of MoS_2_ is beneficial for narrowing the band gap and reducing the recombination rate of photo-generated electrons and holes, and the photocatalytic performance of CNM (1:2) increased. The composite photocatalyst has a wide pH range and can still show a high removal rate after multiple reuses, overcoming the shortcomings of existing difficult-to-recover photocatalysts.

## 2. Results and Discussion

### 2.1. Characterization

#### 2.1.1. XRD

**Figure 1:**
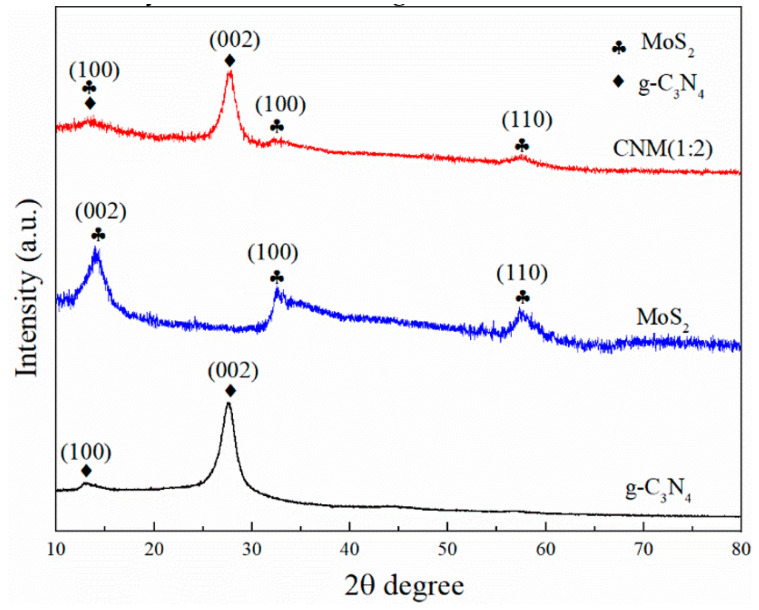
XRD patterns of g-C_3_N_4_, MoS_2_, and CNM (1:2).

The X-ray diffraction (XRD) pattern characterization of g-C_3_N_4_, MoS_2_, and CNM (1:2) is shown in Figure 1. g-C_3_N_4_ had diffraction peaks at 13.1° and 27.6°, which corresponded to the (100) and (002) planes, respectively [45]. The characteristic diffraction peaks of 13.9°, 32.8° and 58.6° were attributed to the (002), (100), and (110) planes, respectively [46]. Compared with g-C_3_N_4_ and MoS_2_, there is a shift in the peak position of the CNM (1:2) composites, which may be caused by the increased interlayer distance of the defect modified samples [47]. The diffraction peaks of the CNM (1:2) composites were 13.3°, 27.9°, 32.8° and 57.6°, respectively. These were attributed to (100), (002), (100), and (110). The CNM (1:2) composites showed diffraction peaks belonging to g-C_3_N_4_ and MoS_2_, which indicated the MoS_2_ had successfully combined with g-C_3_N_4_.

#### 2.1.2. SEM

**Figure 2:**
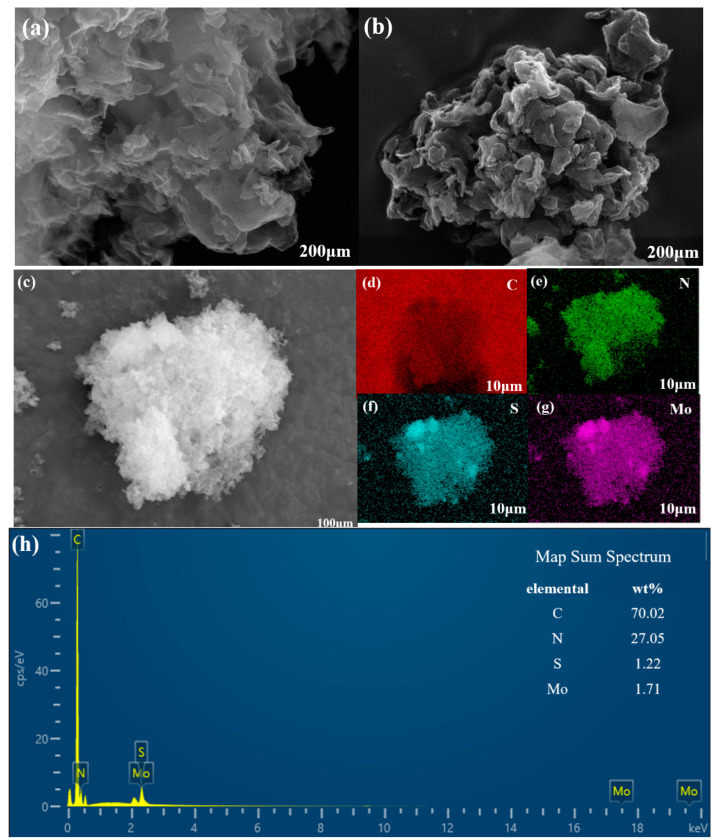
SEM images of samples: (**a**) g-C_3_N_4_, (**b**) CNM (1:2), (**c**) SEM image of CNM (1:2), and corresponding elemental mapping of (**d**) C, (**e**) N, (**f**) S, and (**g**) Mo. (**h**) EDS spectrum of CNM (1:2).

Scanning electron microscopy (SEM) images of g-C_3_N_4_ and g-C_3_N_4_/MoS_2_ are shown in Figure 2. Figure 2a,b show SEM images of g-C_3_N_4_ and CNM (1:2), g-C_3_N_4_ had a massive structure, which was combined with a layered structure. CNM (1:2) had a porous structure, the reason for this result may be that during the ultrasonic treatment, the g-C_3_N_4_ was stripped and combined with MoS_2_, and a large number of pore structures were formed in the process. Ultrasonic treatment can effectively promote g-C_3_N_4_ and MoS_2_ recombination, effectively accelerate the separation of photo-generated carriers, and improve the photo-generated carrier migration rate, thus improving the photocatalytic activity of composite photocatalytic materials. The CNM (1:2) complex can accelerate the separation of photo-generated carriers and increase the migration rate of photo-generated carriers, thus improving the photocatalytic activity of the composite photocatalyst. Figure 2c–h reveal CNM (1:2) as well as corresponding elemental mapping of C, N, S, and Mo, and EDS of CNM (1:2). The mapping of SEM confirmed that the elements of C, N, S, and Mo exist in CNM (1:2), and it indicated that g-C_3_N_4_ and MoS_2_ had been successfully combined. In the process of MoS_2_ doping g-C_3_N_4_, a g-C_3_N_4_/MoS_2_ photocatalyst with more voids was formed, which increased the area of the photocatalyst in contact with pollutants, and increased the number of active sites on its surface, thus increasing its photocatalytic activity.

#### 2.1.3. XPS

**Figure 3:**
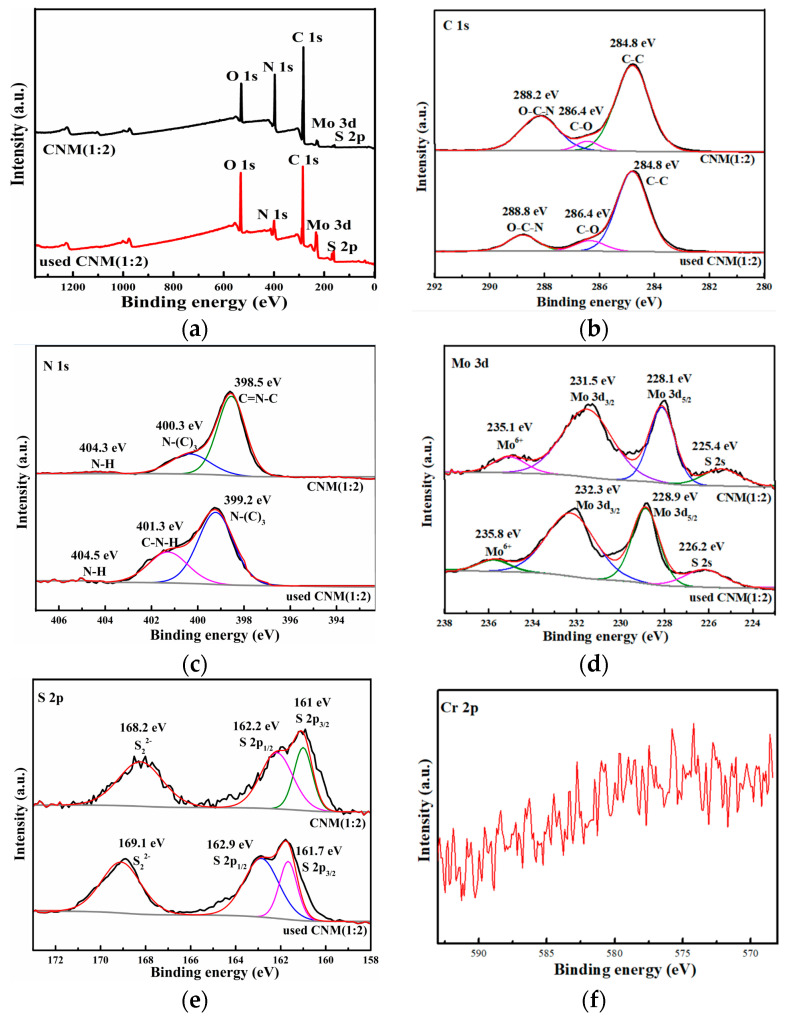
XPS spectra of CNM (1:2) and used CNM (1:2): (**a**) Survey spectra, (**b**) C 1s, (**c**) N 1s, (**d**) Mo 3d, (**e**) S 2p, (**f**) Cr 2p.

The X-ray photoelectron spectroscopy (XPS) spectra of and CNM (1:2) and used CNM (1:2) are displayed in Figure 3, and the spectra of g-C_3_N_4_ were shown in previous articles [30]. The survey spectra of CNM (1:2) and used CNM (1:2) are shown in Figure 3a, the main elements are C, N, O, S and Mo, indicating g-C_3_N_4_ and MoS_2_ were successfully combined. In Figure 3b, the C 1s spectrum of CNM (1:2) has three peaks at 288.2, 286.4, and 284.8 eV; the 288.2 eV is attributed to O-C-N, the 286.4 eV is attributed to C-O, and the 284.8 eV belongs to C-C [48]. In the C 1s spectrum of used CNM (1:2), the peaks shifted to 288.8, 286.4 and 284.8 eV, respectively [49]. The N 1s of CNM (1:2) has three peaks at 404.3, 400.3 and 398.5 eV, which are attributed to N-H bonds, N-(C)_3,_ and C = N-C [50]. Additionally, the N 1s peaks of the used CNM (1:2) were shifted to 404.5 eV (N-H), 401.3 eV (C-N-H), and 399.2 eV (N-(C)_3_) [51], as shown in Figure 3c. The Mo 3d of CNM (1:2) had four peaks at 235.1, 231.5, 228.1 and 225.4 eV, with the peaks corresponding to Mo^6+^, Mo 3d_3/2_, Mo 3d_5/2_ and S 2s, attributed to the 1T-phase MoS_2_ [52,53]. In the Mo 3d spectrum of used CNM (1:2), the peaks shifted to 235.8, 232.3, 228.9, and 226.2 eV, respectively [45]. Figure 3e shows the S 2p spectra of CNM (1:2); it has three peaks at 168.2, 162.2, and 161 eV, which correspond to S_2_^2−^, S 2p_1/2,_ and S 2p_3/2_ [53]. In the S 2p spectrum of used CNM (1:2), those peaks shifted to 169.1, 162.9, and 161.7 eV [45]. In Figure 3f, the presence of Cr was not detected on the surface of the reused photocatalysts, which ensures that the active sites on the surface of the photocatalyst were not covered. The XPS characterization results again confirm that both g-C_3_N_4_ and MoS_2_ have been successfully compounded, and Cr was not detected in the reused CNM (1:2), thus ensuring the excellent reusable performance of the photocatalyst.

#### 2.1.4. BET

**Figure 4:**
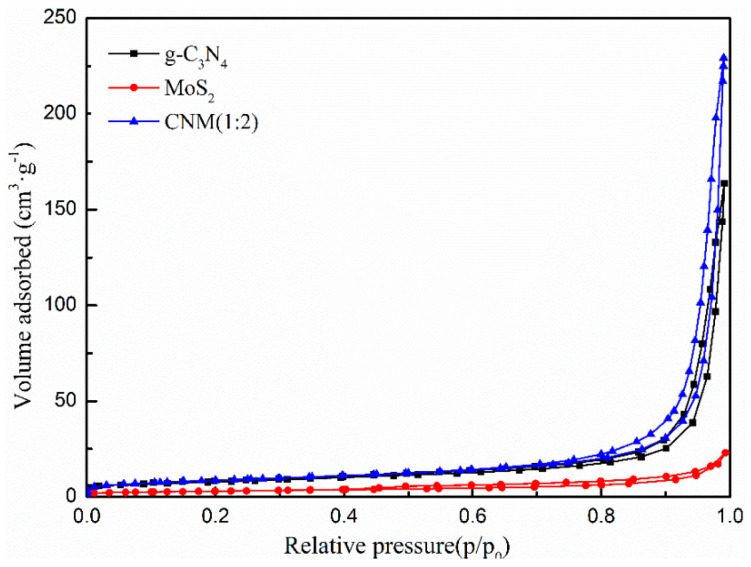
N_2_ adsorption–desorption isotherms of g−C_3_N_4_, MoS_2_ and CNM (1:2).

The Brunauer–Emmett–Teller N_2_ adsorption–desorption isotherms of g-C_3_N_4_, MoS_2_ and CNM (1:2) are shown in Figure 4. The specific surface area and pore volumes of g-C_3_N_4_, MoS_2,_ and CNM (1:2) are listed in Table 1. The specific surface area of CNM (1:2) (30.7214 m^2^·g^−1^) is higher than that of g-C_3_N_4_ (27.3882 m^2^·g^−1^) and MoS_2_ (10.5045 m^2^·g^−1^). In addition, we found that the pore volumes of g-C_3_N_4_ (0.2385 cm^3^·g^−1^) and MoS_2_ (0.0332 cm^3^·g^−1^) are lower than that of CNM (1:2) (0.3498 cm^3^·g^−1^). The increase in specific surface area and pore volume is beneficial to providing more photocatalytic active sites and improving the activity of CNM (1:2).

#### 2.1.5. UV–Vis Diffuse Reflectance Spectra

**Figure 5:**
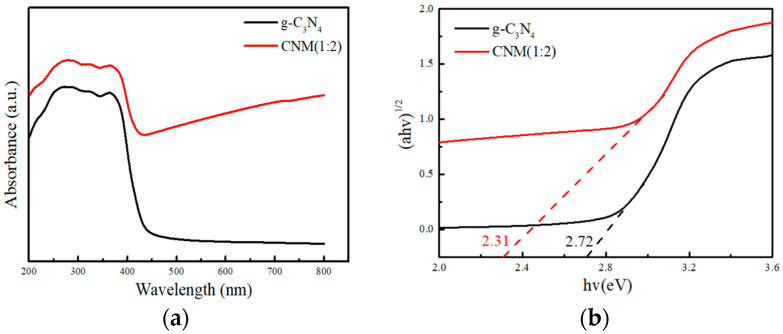
(**a**) UV–vis diffuse reflectance spectra. (**b**) Plots of the transformed Kubelka–Munk function versus the photon energy of g-C_3_N_4_ and CNM (1:2).

UV–vis diffuse reflectance spectra images of g-C_3_N_4_ and CNM (1:2) are displayed in Figure 5a. g-C_3_N_4_ had an absorption edge at 450 nm, while the absorption edge of CNM (1:2) was redshifted, and the absorption range was 200–800 nm. This indicates that the doping of MoS_2_ could effectively improve the utilization ratio of the photocatalyst in visible light, and the light absorption of the photocatalyst was enhanced, thus achieving the purpose of improving the photocatalytic activity of CNM (1:2). The plots of the transformed Kubelka–Munk function versus the photon energy of g-C_3_N_4_ and CNM (1:2) are shown in Figure 5b. The value of the band gap can be calculated according to the formula (ahv)^1/2^ = A(hv−Eg), hv = hc/λ; the results show that the band gaps of g-C_3_N_4_ and CNM (1:2) are 2.72 and 2.31 eV, respectively. The doping of MoS_2_ is beneficial to narrowing the band gap and reducing the recombination rate of photo-generated electrons and holes.

#### 2.1.6. PL Spectra

**Figure 6:**
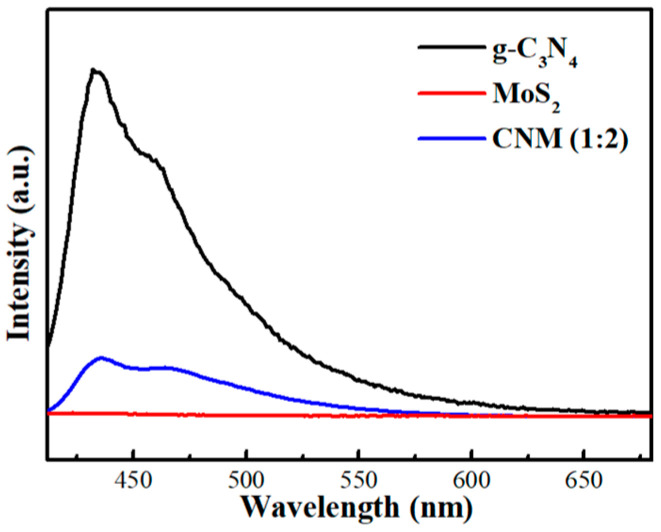
PL spectra of g-C_3_N_4_, MoS_2,_ and CNM (1:2).

The separation and transfer ability of photo-excited carriers in the g-C_3_N_4_, MoS_2_ and CNM (1:2) are measured with PL spectra. As displayed in Figure 6, the g-C_3_N_4_ and CNM (1:2) exhibited broad peaks at 435 nm. g-C_3_N_4_ showed a stronger fluorescence emission peak intensity, which indicates the higher recombination efficiency of the electron–hole pairs. After adding MoS_2_, the CNM (1:2) showed a low PL intensity, the carrier separation efficiency was enhanced, and the electron pairs’ separation rate was reduced in the CNM (1:2) nanocomposite. Therefore, the results confirmed the photocatalytic performance of CNM (1:2) increased.

#### 2.1.7. Transient Photocurrent Responses

**Figure 7:**
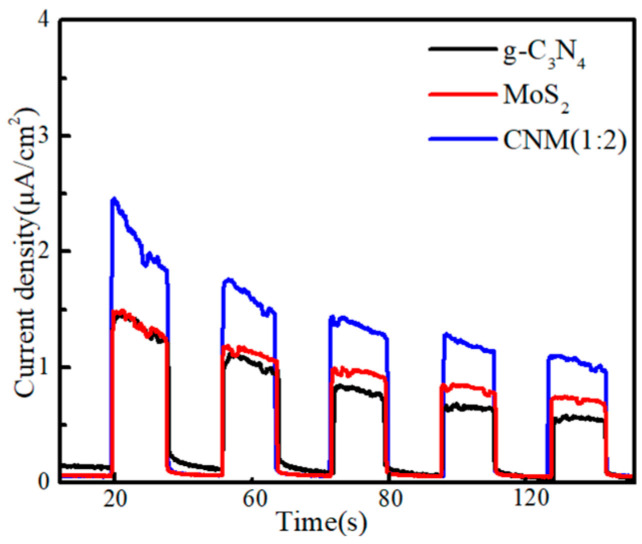
Transient photocurrent responses of g−C_3_N_4_ MoS_2_ and CNM (1:2).

The separation and transfer of photo-excited carriers on g-C_3_N_4_ MoS_2_ and CNM (1:2) were investigated. The transient photocurrent spectrum of the samples is shown in Figure 7. The CNM (1:2) showed the highest photocurrent density during the samples; it showed that the separation efficiency of the photogenerated electrons and holes is significantly improved after the doping MoS_2_. This result confirms that CNM (1:2) can effectively reduce the recombination rate of electrons and holes, thereby increasing the photocatalytic activity of the photocatalysts.

### 2.2. Photocatalysis

#### 2.2.1. Photocatalytic Performance of Different Photocatalysts

**Figure 8:**
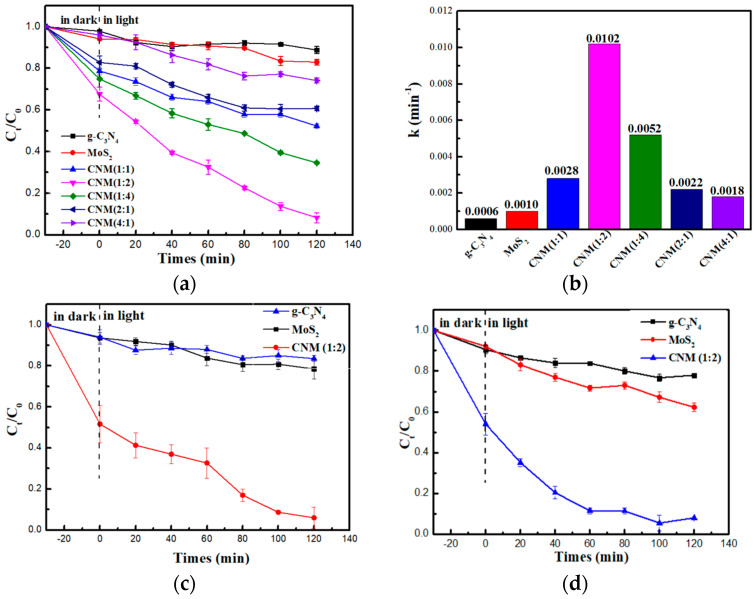

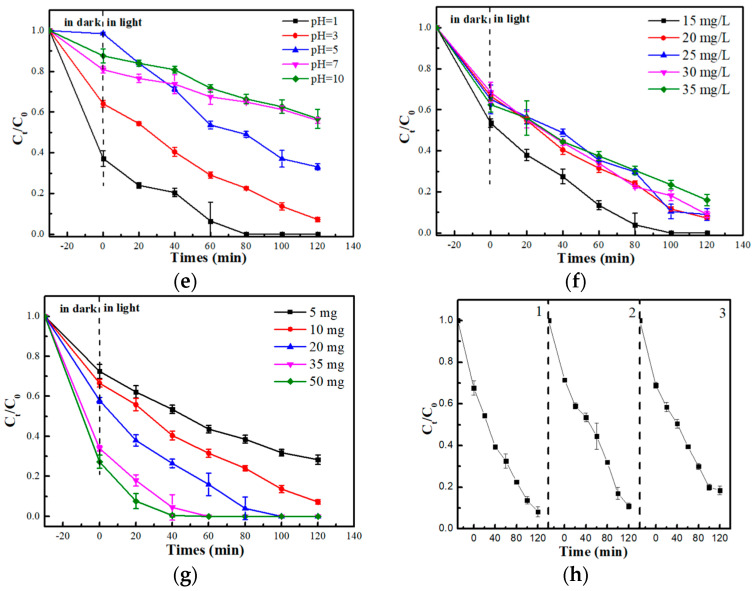
(**a**) The removal of Cr (VI) by different photocatalysts. (**b**) Pseudo−first−order reaction kinetics. (**c**) Photocatalytic removal of Cr (VI) under ultraviolet light. (**d**) Photocatalytic removal of Cr (VI) under solar light. (**e**) Effect of pH. (**f**) Effect of initial concentration. (**g**) Effect of photocatalytic dosage. (**h**) Recyclability of CNM (1:2).

The removal rates and the pseudo-first-order reaction kinetics of Cr (VI) by g-C_3_N_4_, MoS_2_, CNM (1:1), CNM (1:2), CNM (1:4), CNM (2:1), and CNM (4:1) were shown in Figure 8a,b. In order to select the optimal ratio of g and m, we prepared photocatalysts with different mass ratios, as shown in Figure 8a, the results show that doping with different MoS_2_ masses has a great influence on the photocatalytic activity of the photocatalysts. When the mass ratio of g-C_3_N_4_ to MoS_2_ was 2:1 (CNM (1:2)), it had the best rate of removal of Cr (VI). At the same time, the pseudo-first-order reaction kinetics model shows that the reaction rates of g-C_3_N_4_, MoS_2_, CNM (1:1), CNM (1:2), CNM (1:4), CNM (2:1) and CNM (4:1) are, respectively, 0.0006, 0.0010, 0.0028, 0.0102, 0.0052, 0.0022, and 0.0018 min^−1^. It was found that when the content of MoS_2_ was too high, the removal efficiency of Cr (VI) was decreased. Excessive MoS_2_ made the charge transfer rate too fast, which increases the recombination probability of photo-generated electrons and holes; therefore, the photocatalytic activity decreased.

In order to investigate the removal ability of photocatalysts, we explored the Cr (VI) removal rate of photocatalysts under different forms of light illumination (ultraviolet light, solar light, and visible light), and the results displayed in Figure 8c,d. The removal rate of Cr (VI) by CNM (1:2) was 91.6% under the illumination of ultraviolet light, and the removal rate of Cr (VI) was 91% and 86% under solar light and visible light, respectively. The results revealed that with the different forms of light irradiation, the photocatalytic removal performance of Cr (VI) had a weak effect. At the same time, we investigated the effects of pH value, initial concentration, and dosage on the removal rate (in Figure 8e–g), The experimental results show that with the increase in the pH value, the reducibility of the photocatalyst to Cr (VI) decreases gradually. When the solution is neutral or alkaline, the removal rate of Cr (VI) reduced to 40%, but the removal rate is as high as about 65% under weak acid conditions, which affirms that photocatalysts have a high application range. The stability of the photocatalysts was experimentally assessed three times under visible light (in Figure 8h), and the results showed that the CNM (1:2) photocatalysts still had strong photocatalytic activity after multiple cycles. This effectively solves the shortcomings of traditional photocatalysts that can only remove Cr (VI) under strong acid conditions and provides data support for the practical engineering application of photocatalysts in the future.

#### 2.2.2. Scavenging Study

**Figure 9:**
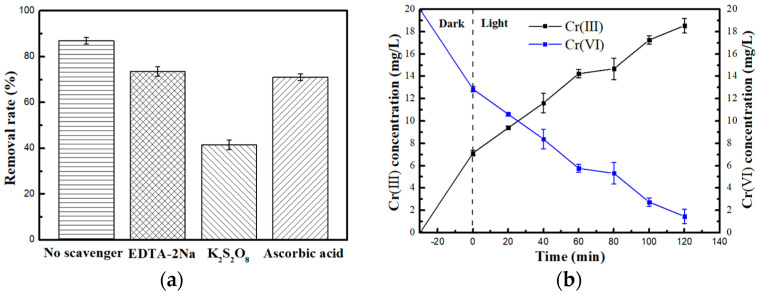
(**a**) The effect of reactive scavengers. (**b**) Variation in Cr valence at different reaction times.

In order to investigate the main reactive radicals in the reactions, scavenger tests were performed and the results are displayed in Figure 9a. In this study, ethylenediaminetetraacetic acid disodium salt (EDTA−2Na), potassium persulfate (K_2_S_2_O_8_), and ascorbic acid (C_6_H_8_O_6_) were used as the scavenger for scavenge holes (h^+^), electrons (e^−^), and superoxide radicals (·O_2_^−^), respectively. The test results showed that the removal of Cr (VI) by the CNM (1:2) photocatalyst was significantly decreased after the addition of K_2_S_2_O_8_, which implied that the e^−^ played an important role in the removal of Cr (VI), and the results displayed that the addition of ascorbic acid and EDTA-2Na have a slight influence on the removal of Cr (VI), which shows that ·O_2_^−^ and h^+^ do not play a key role in the reaction. 

To evaluate the reaction mechanism of CNM (1:2)’s photocatalytic reduction of Cr (VI), an inductively coupled plasma–mass spectrometer (ICP-MS) was used to analyze the concentration of chromium. Ultraviolet-visible spectrophotometry was performed to measure the concentration of Cr (VI), which can determine the valence state change of chromium during the reaction (Figure 9b). The results show that the content of total chromium did not change with the increase in reaction time, but the concentration of Cr (VI.) decreased with the increase in light time. The presence of chromium in aqueous solution mainly includes Cr (III) and Cr (VI), from which it can be inferred that with the increase in reaction time, Cr (VI) in water is reduced to Cr (III). The content of total chromium in the solution remains unchanged, which indicates that the Cr (VI) in the water is reduced to Cr (III) by photocatalysis during the reaction process. Chromium is mainly present in water in the form of minimally toxic Cr (III) instead of being adsorbed on the surface of the photocatalyst; this means it will not form a buildup on the surface of the material and affect the performance of the photocatalyst, which is beneficial for recycling of photocatalysts.

#### 2.2.3. Mechanisms of Photocatalysis

**Figure 10:**
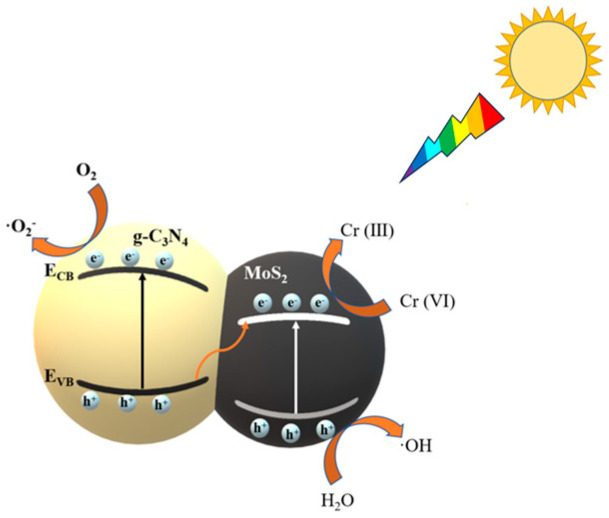
The mechanism for removal of Cr (VI) by CNM (1:2).

A possible photocatalytic mechanism of CNM (1:2) removal Cr (VI) is shown in Figure 10. Additionally, the figure shows that under the excitation of light, a large number of electron and hole pairs are generated on the surface of photocatalysts, which greatly increases the activity of the photocatalyst for pollutant removal [54]. The band gap of CNM (1:2) was significantly narrower than that of the g-C_3_N_4_ and MoS_2_, which is due to the photoexcited electrons’ (e^−^) transition from the conduction band (CB) of g-C_3_N_4_ to the CB of MoS_2_, and the holes’ (h^+^) transition from the valence band (VB) of MoS_2_ to the VB of g-C_3_N_4_, which is beneficial to the narrowing of the band gap and reduces the recombination rate of photo-generated electrons and holes. The photocatalyst can absorb more energy under the same light conditions and be excited to generate more photo-generated electron–hole pairs, thus improving the photocatalytic performance of CNM (1:2) and enhancing the removal rate of Cr (VI) by CNM (1:2). This is due to Z-scheme heterojunction formed between g-C_3_N_4_ and MoS_2_ [44]. The reaction equation is shown in Equations (1)–(4).

g-C_3_N_4_ + hv → g-C_3_N_4_ (e^−^ + h^+^) (1)

g-C_3_N_4_ + MoS_2_ → g-C_3_N_4_ + MoS_2_(e^−^) (2)

MoS_2_ (e^−^) + O_2_ → MoS_2_ + ·O_2_^−^ (3)

Cr (VI) + e^−^ → Cr (Ⅲ) (4)

## 3. Materials and Methods

### 3.1. Materials

Thiourea (H_2_NCSNH_2_, AR) was purchased from Hengxing chemical preparation(Tianjin, China), ammonium molybdate ((NH_4_)_6_Mo_7_O_24_·4H_2_O, AR) was acquired from Taishan Chemical Plant (Shandong, China), urea (H_2_NCONH_2_, AR) was obtained from Tianjin Zhiyuan chemical reagent co(Tianjin, China), ethylenediaminetetraacetic acid disodium salt (EDTA-2Na), potassium persulfate (K_2_S_2_O_8_), ascorbic acid (C_6_H_8_O_6_), hydrochloric acid (HCL), and sodium hydroxide (NaOH) were obtained from Xilong scientific (Guangzhou, China). 

### 3.2. Preparation of Photocatalysts

#### 3.2.1. Preparation of g-C_3_N_4_

g-C_3_N_4_ was prepared by the thermal polymerization method. Then, 20 g of urea were loaded into a crucible and wrapped in tin foil, with a 5 °C·min^−1^ heating rate, before being kept at 550 °C for 4 h. After cooling to room temperature, the yellow g-C_3_N_4_ was obtained, using an agate mortar grind to obtain the powdered g-C_3_N_4_. 

#### 3.2.2. Preparation of MoS_2_

The MoS_2_ was prepared by the hydrothermal method. Then, 0.4 g ammonium molybdate ((NH_4_)_6_Mo_7_O_24_·4H_2_O) and 0.8 g thiourea (H_2_NCSNH_2_) were added to 10 mL of deionized water, stirred for 30 min, and ultrasound-treated for 30 min. The solution was transferred to a hydrothermal reactor and heated for 10 h at 200 °C, after which the solution was cooled to room temperature and strained and washed with deionized water and anhydrous ethanol 3 times, aiming to remove any impurities. The samples were kept at 60 °C for 12 h; the black MoS_2_ was obtained using an agate mortar to grind it into a powder (which was bagged for later use). 

#### 3.2.3. Preparation of g-C_3_N_4_/MoS_2_ with Different Mass Ratios

**Figure 11:**
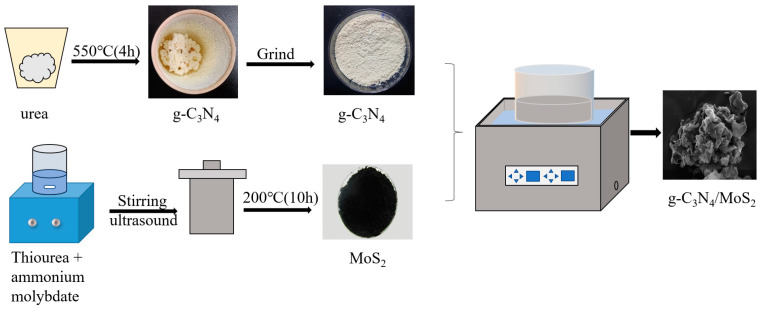
The preparation process of photocatalysts.

g-C_3_N_4_/MoS_2_ with different mass ratios was prepared by the ultrasonic method. The 0.2 g g-C_3_N_4_ and 0.4 g MoS_2_ were added to 400 mL of deionized water and ultrasound-treated for 180 min. The g-C_3_N_4_/MoS_2_ was obtained and denoted CNM (1:2); the CNM (1:4), CNM (2:1), CNM (4:1), and CNM (1:1) were obtained in the same way, and the different samples were obtained by changing the mass ratios of g-C_3_N_4_ and MoS_2_, as shown in Figure 11. 

### 3.3. Characterization of Photocatalysts

The crystal structures of the photocatalysts were characterized by X-ray diffraction (XRD, Rigaku SmartLab SE, Rigaku Corp., Tokyo, Japan) with Cu-kα radiation. The morphology and structure of the photocatalysts were determined using scanning electron microscopy (SEM, TESCAN MIRA LMS, TESCAN CHINA, Ltd., Shanghai, China). X-ray photoelectron spectroscopy (XPS, Thermo Scientific k-Alpha, Thermo Fisher Scientific Co., Ltd., Shanghai, China) was used to characterize the elemental composition and valence state of the photocatalysts. The optical properties of photocatalysts were measured using a UV–visible spectrophotometer (UV–vis, UV-3600i plus, Shimadzu Corp., Kyoto, Japan). The surface area and pore size of photocatalysts were tested by the Brunauer–Emmett–Teller method (BET, Micromeritics ASAP2460, Micromeritics Corp., Norcross, GA, USA). The steady and transient photoluminescence spectra of photocatalysts were assessed using a fluorescence spectrometer (PL, FLS980, Edinburgh Instruments Ltd., Shanghai, China). The metal element content was tested using an inductively coupled plasma–mass spectrometer (ICP-MS, PerkinElmer NexION 2000, Perkinelmer, MS, USA).

### 3.4. Photocatalytic Tests

The photocatalytic performance of g-C_3_N_4_, CNM (1:2), CNM (1:4), CNM (2:1), CNM (4:1) and CNM (1:1) was tested in the photoreactor. A 300 W Xe lamp supported by CEL-LAM with a cutoff filter (λ > 420 nm) was used as the source of solar light and visible light. CEL-LAM 500 was the source of UV light, and the reaction took place at room temperature. A 10 mg photocatalyst was put into the 100 mL Cr (VI) solution (20 mg·L^−1^), and the pH value was 3. The photocatalyst and the 20 mg·L^−1^ Cr (VI) solution were stirred in the dark for 30 min to achieve adsorption–desorption equilibrium, and 2 mL of solution was taken each time to analyze the concentration of Cr (VI). After turning on the light source, samples were taken at 20, 40, 60, 80, 100, and 120 min, respectively. The same 2 mL of solution was taken at a time and filtered using a biofilter membrane (0.45 μm) to obtain a filtrate without photocatalyst. We then determined the water quality and amount of chromium (VI)-1,5 dtphenylcarbohydrazide using a spectrophotometric method (GB 7467-87) [55] to analyze the concentration of hexavalent chromium. The removal rate (%) was calculated according to Equation (5):

(5) $$\mathrm{removal}\;\mathrm{rate}\;(\%)=\frac{C_{0}-C_{t}}{C_{0}}\times 100\%$$

*C*_0_: the initial concentration of Cr (VI); *C_t_*: the concentration of Cr (VI) at the corresponding time.

## 4. Conclusions

Z-scheme g-C_3_N_4_/MoS_2_ nanocomposite heterojunctions were successfully synthesized using an ultrasonic method and demonstrated efficient photocatalytic removal of toxic Cr (VI) from water under UV, visible, and solar light irradiation. The nanocomposites, especially those with the optimized 1:2 g-C_3_N_4_/MoS_2_ ratio, exhibited enhanced photoactivity compared to pure g-C_3_N_4_, with over 90% Cr (VI) removal achieved. The superior performance is attributed to the combined effects of the narrowed heterostructure band gap, which enables visible light response, and the effective separation of photo-generated electron–hole pairs at the interfaced junction between the two semiconductors. The results showed that the MoS_2_ could be located at the g-C_3_N_4_, which was beneficial for the enhancement of photocatalytic activity, owing to the g-C_3_N_4_/MoS_2_ nanocomposites having a broad range of light response and the separation and transfer efficiencies of photo-generated electron–hole pairs being improved. Overall, this work highlights the promise of ultrasonically synthesized g-C_3_N_4_/MoS_2_ nanocomposites for tackling the pressing environmental challenge of heavy metal wastewater treatment using solar-driven photocatalysis. Further optimization to translate this efficient lab-scale Cr (VI) remediation to real-world applications should be pursued.

## Figures and Tables

**Table 1:** Specific surface area and pore volumes of g-C_3_N_4_, MoS_2,_ and CNM (1:2).

Samples	S_BET_ (m^2^·g^−1^)	V_Pore_ (cm^3^·g^−1^)
g-C_3_N_4_	27.3882	0.2385
MoS_2_	10.5045	0.0332
CNM (1:2)	30.7214	0.3498

## Data Availability

The data presented in this study are available upon request from the corresponding author. The data are not publicly available due to ethical considerations.
